# Impact of COVID-19 on patient follow-up during supportive periodontal therapy: a retrospective study based on phone call survey

**DOI:** 10.1186/s12903-023-03539-1

**Published:** 2023-10-28

**Authors:** Mengli Wang, Yuxin Xu, Wen Fang, Weiyi Pan, Qianting Wang

**Affiliations:** Stomatology Hospital, School of Stomatology, Zhejiang University School of Medicine, Zhejiang Provincial Clinical Research Center for Oral Diseases, Key Laboratory of Oral Biomedical Research of Zhejiang Province, Cancer Center of Zhejiang University, Hangzhou, Zhejiang 310000 China

**Keywords:** Periodontitis, COVID-19, Loss to follow-up, Retrospective study, Phone-call survey

## Abstract

**Background:**

COVID-19 and the subsequent intermittent lockdown measures from 2020 to 2022 in China critically disrupted regular medical activities, including dental care. This study aimed to investigate the impact of COVID-19 on long-term follow-up at the Stomatology Hospital, Zhejiang University School of Medicine and to evaluate potential causes of loss to follow-up.

**Methods:**

A total of 1062 patients with periodontitis who visited the hospital from January 2019 to June 2022 were included in this study, and patient information was collected retrospectively in the form of a telephone questionnaire. The questionnaire consisted of 19 questions in four areas: demographic characteristics, clinical periodontal parameters, oral hygiene habits, and follow-up-related open-ended questions (specific reasons for loss to follow-up, attitudes toward follow-up and suggestions for increasing participation in future follow-ups). Regression analysis of factors influencing the follow-up of patients with periodontitis were analyzed by regression analysis using R (v4.2.3) software.

**Results:**

A total of 536 (50.47%) valid questionnaires were collected from 1062 patients. Personal factors (42.5%), instead of the COVID-19 epidemic (20.0%), were the main factors that impacted the loss to follow-up in long-term periodontal treatment, while work factors (19.8%), hospital factors (16.4%), and transportation or distance factors (14.7%) were all important factors. A family history of periodontitis [odds ratio (OR) = 0.567, 95% confidence interval (CI): 0.393, 0.817, *p* = 0.002], as well as frequent use of dental devices (OR = 0.540, 95% CI: 0.375, 0.777, *p* = 0.001), were significantly associated with a “negative” attitude toward follow-up visits.

**Conclusion:**

This survey suggests that the COVID-19 epidemic factor was an important cause contributed to the loss to follow-up during supportive periodontal therapy (SPT) among a variety of potential factors. Majority of patients had negative attitudes toward subsequent continued participation in supportive care.

**Supplementary Information:**

The online version contains supplementary material available at 10.1186/s12903-023-03539-1.

## Background

 Periodontitis is the primary cause of adult tooth extractions in China [1]. Patients with periodontitis usually suffer from bleeding gums and loss of periodontium support; this is manifested by a loss of clinical attachment and the presence of periodontal pockets and alveolar bone resorption on radiographs [2]. In severe cases, periodontitis can result in loosening and loss of teeth, which can in turn lead to impaired speech and chewing functions, affecting patients’ physical appearance and having a negative impact on their nutrition, quality of life and mental health [3–6]. A thorough treatment plan is therefore needed for patients with periodontitis to avoid these conditions. The treatment procedure for periodontitis consists of the following four stages: initial therapy, periodontal surgery, restorative therapy, and supportive periodontal therapy (SPT) [1]. SPT is an integral part of a systemic periodontal treatment plan and is a prerequisite for the long-term maintenance of periodontal efficacy [7, 8]. Individually scheduled SPT based on the specific patient’s risk profile needs to be started immediately after initial therapy, and it requires consistent patient participation for the whole treatment duration thereafter [9].

Long-term periodontal health maintenance largely depends on regular and frequent check-ups; unfortunately, many patients are lost to follow-up after active therapy [10–12] despite dentists’ repeated emphasis on the importance and necessity of follow-up in long-term SPT. Previous studies have suggested that age, severity of periodontitis, periodontal surgery and the level of self-efficacy for self-care might be effective predictors of loss to follow-up in long-term SPT among patients with periodontitis [10, 13, 14]. COVID-19 and subsequent intermittent lockdown measures disrupted dental visits, during which dental offices were closed and patient access became more complicated. We hypothesized that COVID-19 might have an impact on loss to follow-up among patients with periodontitis.

Therefore, this study aimed to investigate the impact of COVID-19 on the long-term follow-up among periodontal patients and to evaluate potential causes of loss to follow-up.

### Ethics and study design

This study consisted of a questionnaire survey of the loss to follow-up using telephone callbacks, and the purpose of the trial was clearly explained to the patients to obtain their verbal consent before the questionnaire was administered. After the survey, the researchers answered other inquiries from the patients. This study was approved by the Ethics Committee of the Stomatology Hospital at the School of Stomatology of the Zhejiang University School of Medicine (IP 2022(041)) and was conducted in accordance with the Declaration of Helsinki, as revised in 2013.

#### Telephone questionnaire

The questionnaire used in this survey was developed for this study. The process of development consisted of a literature review study, two group discussions and a telephone questionnaire test with 18 randomly selected patients with periodontitis. It consisted two parts: data collection form and telephone questionnaire. The part one data collection form which including gender, age and clinical periodontal parameters was collected and filled by Pan, W.Y. Telephone surveys were conducted by Xu, Y.X. and Wang, M.L. both of whom had undergone standardized training. Questions 11, 12, and 13 of Part 2 were asked in an open-ended manner. The specific details of the content for the question are presented in Table 1. The specific reasons for failure to attend follow-up visits were discussed by Wang, M.L. and Xu, Y.X. and reviewed by Wang, Q.T. before being classified into 11 factors. The explanation of the classification of each factor is presented in Supplementary file 1.

**Table 1:** Questionnaire

Dear Sir/Madam! Are you aware that you have periodontitis? You are diagnosed with periodontitis by your dentist, which is a chronic disease of the oral cavity and requires regular follow-up visits. However, you didn't follow up after the treatment. We are now conducting a telephone survey to find out the specific reasons for your loss to follow-up during supportive periodontal therapy (SPT). Here are some relevant questions and it might take your few minutes.		
**Patient ID:**		
**Part 1: Data collection form (filled by the researcher)**		
NO.	Item	Answer
**1**	**Age**	
**2**	**Gender**	
**3**	**Mean PD (mm)**	
**4**	**Mean CAL (mm)**	
**5**	**BOP (%)**	
**6**	**Stage of periodontitis**	A. I (A, B and C) B. II (A, B and C) C. III (A, B and C) D. IV (A, B and C)
**Part 2: Telephone Questionnaire**		
NO.	Questions	Answer
1	**What is your current occupation?**	A. Unemployed or enrolled B. Employed C. Retired
2	**What is your education level?**	A. Less than college degree B. College degree or above C. Unwilling to disclose
3	**Do you have a family history of periodontitis?**	A. Yes B. No
4	**Smoking habits: Do you smoke? How often do you smoke?**	A. Have quit smoking or never smoked B. Smoke occasionally C. Smoke every day
5	**Drinking habits: Do you drink alcohol? How often do you drink alcohol?**	A. Total abstention or never drink B. Moderate alcohol consumption C. Excessive drinking
6	**Do you have a health insurance?**	A. Not insured B. Insured
7	**What is the is the range of monthly income of the family (yuan)?**	A. < 5000RMB B. 5000 ~ 10000RMB C. ≥ 10,000RMB D. Unwilling to disclose
8	**How frequently do you brush your teeth in a day?**	A. ≤ 1 time B. ≥ 2 times
9	**What is your duration of each brushing?**	A. < 3 min B. ≥ 3 min
10	**Whether dental devices (dental flosses, water irrigators, etc.) are regularly used?**	A. Yes B. No
11	**Can you talk about the reasons with me about your loss to follow-up during the SPT?**	
12	**How important do you think it is to actively follow up during the SPT? Will you attend the follow-up more actively in the future?**	
13	**What do you think would help you to be more involved in follow-up visits? Can you make any suggestions?**	

## Materials and methods

### Study population

The information of 16,385 patients visited the Periodontics Department of the Stomatology Hospital, Zhejiang University School of Medicine who underwent supragingival scaling from January 2019 to June 2022 were collected. The patients initial visit of the two senior periodontists were then extracted, comprising a total of 2883. Patients were reviewed in conjunction with their medical records, and a total of 1062 patients were included based on the inclusion and exclusion criteria. In this study, SPT compliance was defined as a patient attending repeated SPT appointments within 6 ± 1 months, whereas appointments that took place more than 6 ± 1 months apart were classified as loss to follow-up.

### Eligibility criteria

Patients who met the following criteria were included: (a) ≥ 18 years old, (b) diagnosis of periodontitis: interdental clinical attachment loss (CAL) is detectable at ≥ 2 non-adjacent teeth, or buccal or oral CAL ≥ 3 mm with probing pocket depth (PD) > 3 mm is detectable at ≥ 2 teeth [15], (c) underwent full-mouth subgingival instrumentation, (d) no follow-up visits within 6 ± 1 months of completion of treatment, and (e) complete set of information present in the patient’s medical records, along with panoramic oral film and electronic periodontal record sheet. The exclusion criteria consisted of (a) having undergone periodontal surgery, (b) patients who clearly expressed their unwillingness to participate in the program during the telephone survey, and (c) patients who did not answer after 3 separate calls (first call, 30 min later, any time the following day).

### Statistical analysis

Data were analyzed using R Version 4.2.3. A *p* value of < 0.05 was considered statistically significant in all analyses. Continuous variables are expressed as means ± standard deviations, and a t test was used for comparison between groups; categorical variables are expressed as frequencies (composition ratios), and a χ2 test was used for comparison between groups. For variables that did not meet the application conditions of the χ2 test, Fisher’s exact probability method was used. Patients were divided into two groups, those with “positive” and “negative” visits, based on their attitudes toward follow-up. Regression analysis was performed using a binary logistic regression model, with attitudes toward follow-up as the dependent variable; the model included 16 factors, such as gender, age, and mean PD, as independent variables to analyze the factors influencing the “negative” attitude toward follow-up visits and were expressed as ratios (ORs) and their 95% confidence intervals (95% CIs).

## Results

**Fig. 1:**
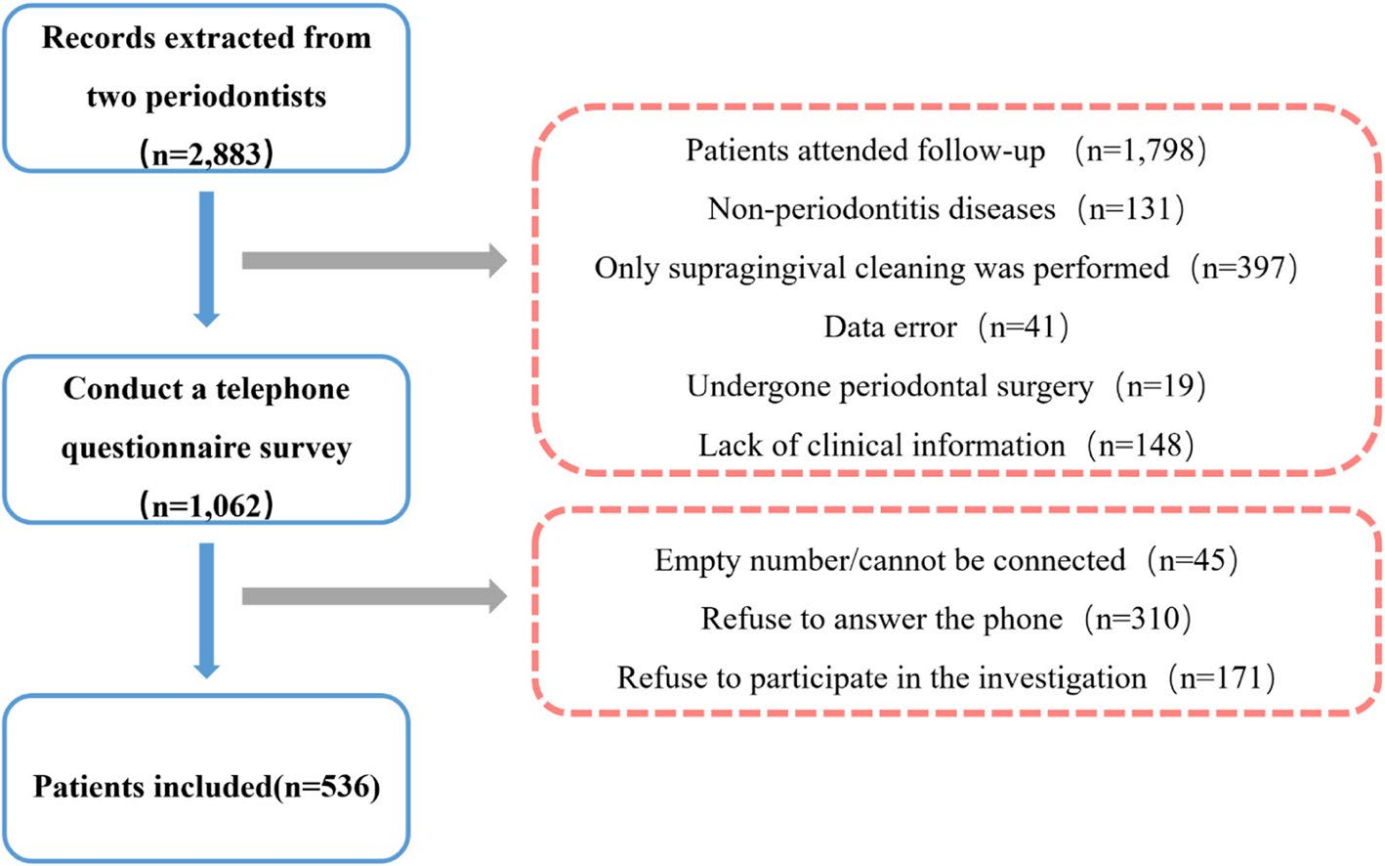
Flowchart of the screening process

A total of 536 valid data points were obtained from 270 (50.4%) males and 266 (49.6%) females, with an effective rate of 50.47%. 45 patients whose calls could not be connected, 310 patients who refused to answer the phone, and 171 patients who refused to participate in this study after being connected were excluded. The specific screening process is shown in Fig. 1.

### Characteristics of the study population

The average age of the participants was 42.1 ± 11.4 years old. The periodontal clinical data of all participants had a mean PD of 3.4 ± 0.8 mm, a mean CAL of 3.5 ± 0.9 mm, and a BOP of 63.9 (20.3%); participants with periodontitis at stage III and IV accounted for 69.9%. Gender, age, occupation, education level, family history of periodontitis, smoking habits, drinking habits, health insurance, income of family per month (yuan), and oral hygiene habits are described in detail in Table 2.

**Table 2:** Characteristics of the study population and general analysis of the attitudes of the two groups towards the follow-up visit

Variables		Follow-up attitude		Overall	*t*/*χ*^2^	*P* value
		Positive	Negative			
Overall		324 (60.4%)	212 (39.6%)	536 (100.0%)		
Age^a^		42.0 ± 11.0	42.2 ± 12.1	42.1 ± 11.4	0.248	0.805
Gender	Male	160 (59.3%)	110 (40.7%)	270 (50.4%)	0.321	0.571
	Female	164 (61.7%)	102 (38.3%)	266 (49.6%)		
Mean PD (mm)^a^		3.4 ± 0.8	3.4 ± 0.8	3.4 ± 0.8	0.069	0.945
Mean CAL (mm)^a^		3.5 ± 0.9	3.5 ± 0.8	3.5 ± 0.9	0.028	0.978
BOP(%)^a^		63.2 ± 20.5	64.9 ± 20.1	63.9 ± 20.3	0.946	0.345
Stage of periodontitis	I (A, B and C)	43 (55.8%)	34 (44.2%)	77 (14.4%)	0.868	0.833
	II (A, B and C)	51 (60.0%)	34 (40.0%)	85 (15.9%)		
	III (A, B and C)	199 (61.4%)	125 (38.6%)	324 (60.4%)		
	IV (A, B and C)	31 (62.0%)	19 (38.0%)	50 (9.3%)		
Occupation	Unemployed or enrolled	12 (54.5%)	10 (45.5%)	22 (4.1%)	0.394	0.821
	Employed	259 (60.9%)	166 (39.1%)	425 (79.3%)		
	Retired	53 (59.6%)	36 (40.4%)	89 (16.6%)		
Education level	Less than college degree	118 (59.6%)	80 (40.4%)	198 (36.9%)	5.750	0.056
	College degree or above	172 (64.2%)	96 (35.8%)	268 (50.0%)		
	Unwilling to disclose	34 (48.6%)	36 (51.4%)	70 (13.1%)		
Family history of periodontitis	No	180 (55.0%)	147 (45.0%)	327 (61.2%)	9.726	0.002**
	Yes	142 (68.6%)	65 (31.4%)	207 (38.8%)		
Smoking habits	Have quit smoking or never smoked	288 (60.6%)	187 (39.4%)	475 (88.6%)	0.649	0.723
	Smoke occasionally	11 (52.4%)	10 (47.6%)	21 (3.9%)		
	Smoke every day	25 (62.5%)	15 (37.5%)	40 (7.5%)		
Drinking habits	Total abstention or never drink	266 (59.6%)	180 (40.4%)	446 (83.2%)	—	0.293^b^
	Moderate alcohol consumption	54 (66.7%)	27 (33.3%)	81 (15.1%)		
	Excessive drinking	4 (44.4%)	5 (55.6%)	9 (1.7%)		
Health insurance	Not insured	25 (50.0%)	25 (50.0%)	50 (9.3%)	2.518	0.113
	Insured	299 (61.5%)	187 (38.5%)	486 (90.7%)		
Income of family per month (yuan)	≤ 5000 RMB	38 (55.9%)	30 (44.1%)	68 (12.7%)	11.526	0.009**
	5000 ~ 10000RMB	106 (61.6%)	66 (38.4%)	172 (32.1%)		
	≥ 10,000 RMB	134 (67.3%)	65 (32.7%)	199 (37.1%)		
	Unwilling to disclose	46 (47.4%)	51 (52.6%)	97 (18.1%)		
Daily tooth brushing frequency	≤ 1 time	18 (58.1%)	13 (41.9%)	31 (5.8%)	0.078	0.780
	≥ 2 times	306 (60.6%)	199 (39.4%)	505 (94.2%)		
Duration of each brushing	< 3 min	105 (59.3%)	72 (40.7%)	177 (33.0%)	0.141	0.708
	≥ 3 min	219 (61.0%)	140 (39.0%)	359 (67.0%)		
Whether dental devices were regularly used	No	91 (50.6%)	89 (49.4%)	180 (33.6%)	11.092	< 0.001**
	Yes	233 (65.4%)	123 (34.6%)	356 (66.4%)		

The stage of periodontitis was classified according to the new AAP/EFP consensus. E.g., I (A, B and C) represent periodontitis (stage I, level A), (stage I, level B) and (stage I, level C)

*PD* Pocket depth, *CAL* Clinical attachment loss, *BOP* Bleeding on probing

**p < 0.05

^a^ Indicate these are continuous variables expressed as means ± standard deviations; the rest are categorical variables expressed as frequencies (composition ratios)

^b^ Fisher exact test

### Reasons for loss to follow-up

**Fig. 2:**
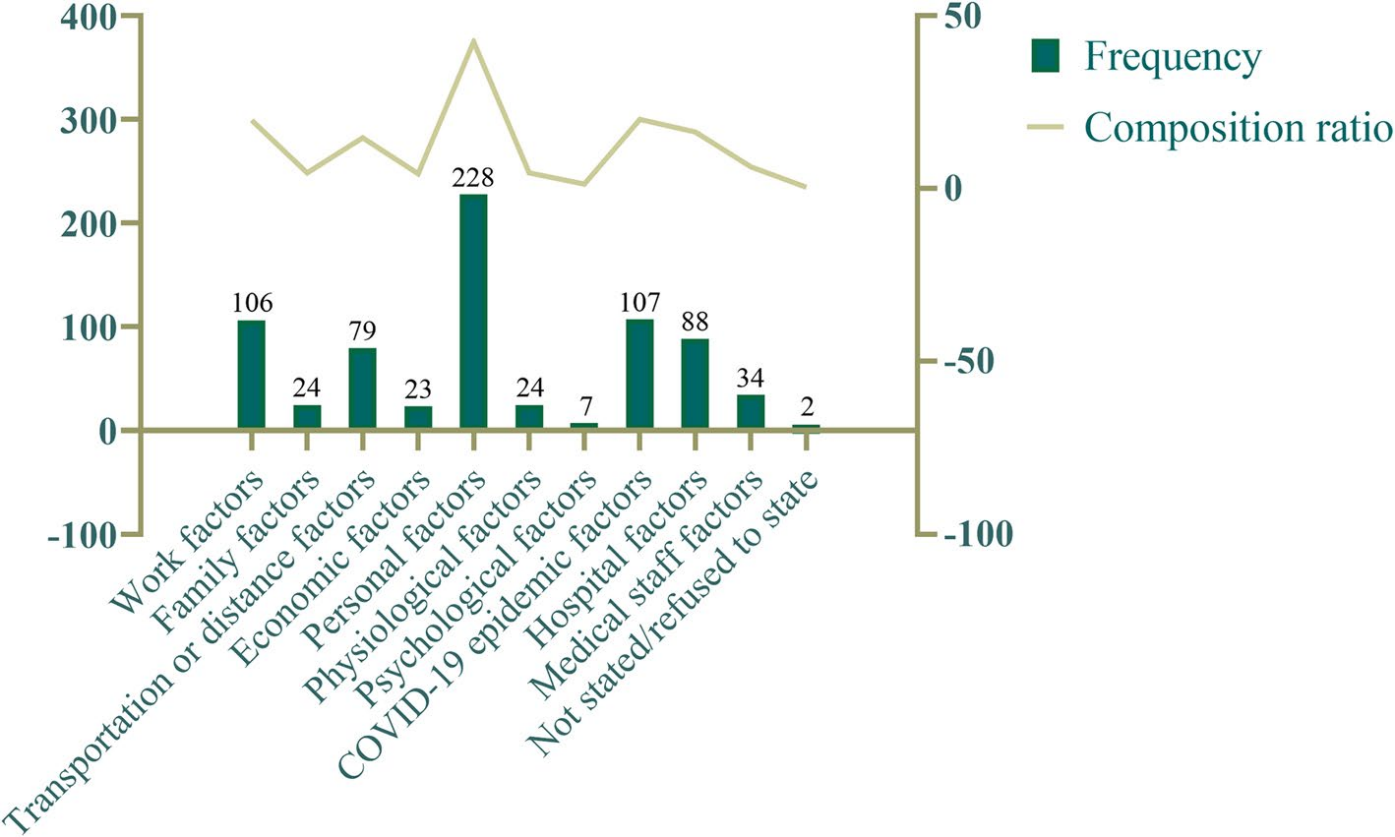
Descriptive analysis of reasons for loss to follow-ups

The specific reasons for failure to attend follow-up visits were varied. The top three factors were personal factors, COVID-19 epidemic factors and work factors, as shown in Fig. 2.

### Attitudes toward follow-up visits and related influencing factors

324 patients (60.4%) held a positive attitude toward follow-up visits, while 212 patients (39.6%) believed that they would not attend any follow-up visits at the periodontal department. To investigate which factors had an impact on patients’ attitudes toward treatment at a later time, a univariate analysis of the included variables was conducted, as shown in Table 2. In the univariate correlation model, 3 independent variables emerged as significant predictors of required periodontal treatment: a family history of periodontitis, family income per month (yuan), and whether dental devices (e.g., dental floss and water irrigator) were regularly used (*p* < 0.05).

All the variables mentioned above were included in a binary logistic regression analysis (Table 3), while family history of periodontitis (OR = 0.567, 95% CI: 0.393, 0.817) and frequent use of dental devices (OR = 0.540, 95% CI: 0.375, 0.777) were negatively correlated with patients’ attitudes toward follow-up visits.

**Table 3:** Multivariate Logistic regression analysis of different attitudes to follow-up

Variables		*β*		*SE*	Wald *χ*^2^	*P*	*OR*(95%*CI*)
Age		0.002	0.008		0.253	0.800	1.002 (0.987, 1.017)
Gender	Male	Reference					
	Female	-0.100	0.177		-0.567	0.571	0.905 (0.640, 1.279)
Mean PD (mm)		-0.008	0.111		-0.069	0.945	0.992 (0.799, 1.232)
Mean CAL (mm)		0.003	0.102		0.027	0.978	1.003 (0.821, 1.225)
BOP(%)		0.004	0.004		0.943	0.346	1.004 (0.996, 1.013)
Stage of periodontitis	I (A, B and C)	Reference					
	II (A, B and C)	-0.171	0.319		-0.535	0.593	0.843 (0.451, 1.575)
	III (A, B and C)	-0.230	0.256		-0.898	0.369	0.794 (0.481, 1.313)
	IV (A, B and C)	-0.255	0.371		-0.687	0.492	0.775 (0.375, 1.604)
Occupation	Unemployed or enrolled	Reference					
	Employed	-0.263	0.440		-0.597	0.550	0.769 (0.325, 1.820)
	Retired	-0.204	0.480		-0.426	0.670	0.815 (0.318, 2.087)
Education level	Less than college degree	Reference					
	College degree or above	-0.194	0.193		-1.008	0.313	0.823 (0.564, 1.201)
	Unwilling to disclose	0.446	0.280		1.595	0.111	1.562 (0.903, 2.701)
Family history of periodontitis	No	Reference					
	Yes	-0.568	0.186		-3.047	0.002**	0.567 (0.393, 0.817)
Smoking habits	Have quit smoking or never smoked	Reference					
	Smoke occasionally	0.337	0.447		0.753	0.451	1.400 (0.583, 3.362)
	Smoke every day	-0.079	0.340		-0.232	0.816	0.924 (0.475, 1.799)
Drinking habits	Total abstention or never drink	Reference					
	Moderate alcohol consumption	-0.303	0.255		-1.188	0.235	0.739 (0.449, 1.217)
	Excessive drinking	0.614	0.678		0.906	0.365	1.847 (0.489, 6.973)
Health insurance	Insured	Reference					
	Not insured	0.469	0.298		1.576	0.115	1.599 (0.892, 2.866)
Income of family per month (yuan)	≤ 5000 RMB	Reference					
	5000 ~ 10,000 RMB	-0.237	0.290		-0.818	0.413	0.789 (0.447, 1.393)
	≥ 10,000 RMB	-0.487	0.287		-1.696	0.090	0.614 (0.350, 1.079)
	Unwilling to disclose	0.340	0.318		1.069	0.285	1.404 (0.753, 2.618)
Daily tooth brushing frequency	≤ 1 time	Reference					
	≥ 2 times	-0.105	0.375		-0.279	0.780	0.900 (0.432, 1.879)
Duration of each brushing	< 3 min	Reference					
	≥ 3 min	-0.070	0.187		-0.374	0.708	0.932 (0.646, 1.346)
Whether dental devices were regularly used	No	Reference					
	Yes	-0.617	0.186		-3.313	0.001**	0.540 (0.375, 0.777)

**p < 0.05

## Discussion

Failure to attend follow-up visits among patients with periodontitis has a negative impact on adherence to good periodontal health after successful periodontal treatment [16]. The present study adopted open-ended questions to allow participants to self-report their particular situation to analyze the specific reasons and related influences for loss to follow-up in depth.

The reasons for loss to follow-up are varied. A total of 381 patients (71.7%) self-reported not attending follow-up appointments due to a single factor, while the remaining 28.9% did not attend due to multiple factors. Personal factors were the most important reasons for loss to follow-up. Patients felt that they had good periodontal health, forgot the time of the follow-up visit, or subjectively avoided treatment. It is evident that patients’ periodontal health awareness is still very inadequate. It has been documented that chief complaints are associated with patient compliance and that acute symptoms may be a positive predictor of periodontal treatment initiation but may be a negative predictor of treatment completion [17]. This view was confirmed in the present study, in which the majority of patients reported that they felt asymptomatic with regard to periodontitis after treatment, which to some extent confirmed the treatment effect. At the same time, since patients often forget to attend appointments (due to the long-time intervals between visits), health care professionals need to provide more ways to remind them to see the doctor. For example, the development and improvement of follow-up reminder systems may have a positive effect on improving appointment adherence, as the literature shows with applications in the orthodontic field [18]. Some patients were subjectively reluctant to receive treatment, stating that they were aware of their doctor’s recommendations for follow-up but still refused to attend appointments on time. Based on previous research, patients suffering from a lack of information and motivation are by far the leading cause of poor adherence, as reported by patients themselves [11]. Therefore, in addition to reminding patients not to miss follow-up visits due to personal factors, the most important thing is to provide them with the corresponding health education, that is, to reinforce the concept outside the clinic to increase their motivation to participate in treatment [18].

The main objective of this study was to investigate the effect of COVID-19 on the long-term follow-up among periodontal patients, which, as shown by the results, accounted for the second reason for loss to follow-up (20.0%), with patients stating that their fear of infection and subsequent intermittent lockdown measures made it difficult for them to move forward, which is a global phenomenon [19]. COVID-19 is now classified as a Class B infectious disease, and this barrier can be removed. 79 (14.7%) of the patients felt that the hospital was not close enough to a subway station, and a significant proportion of patients from out of town were unable to maintain regular follow-ups due to the distance. Both of these factors can be aided by teledentistry. In the last few years, teledentistry has evolved to such an extent that it is able to improve medical efficiency by reducing geographical barriers and the risk of infectious diseases [20, 21]. Teledentistry is an excellent way to provide health education, patient follow-up management, etc. It is therefore important to continuously strengthen the construction and promotion of internet-based applications in hospitals. An increased prevalence of teledentistry to connect specialist dental hospitals with community hospitals or dental clinics for basic review and intelligent referral can, to a certain extent, solve the problem of distance and congestion at the dental hospital. However, China’s current dental specialty institution information technology investment scale is small, and the service level of each dental hospital varies; thus, a more sustainable development path needs to be explored [22].

A total of 106 patients (19.8%) were unable to attend follow-up visits due to work. 88 (16.4%) of the patients abandoned further visits due to hospital factors, such as difficulty in making appointments with periodontists and long waiting times. Both of these objective factors can be explained by the fact that the number of private dental institutions in China is currently twice that of public institutions. The ratio of the number of dentists to the population is 1:7768, which is lower than the WHO standard of 1:5000. The overall number of oral/dental staff and institutions is insufficient and unevenly distributed [23, 24], but most people in China tend to seek treatment at public institutions [25]. Medical resources for public oral/dental institutions are always in short supply, and the treatment of periodontitis requires multiple visits, which inevitably leads to longer waiting times or an inability to provide more doctors on weekends. China rolled out a series of policies in 2015–2016 to establish medical alliances to integrate resources at all levels, expand the scale of private hospitals and relax requirements for doctors to practice at a single location. However, private health care is expensive and caters to only a fraction of patients [26]. This is a very difficult issue. We have the following suggestions: We have the following suggestions: public hospitals can try to open more branches to cover more districts and alleviate the burden on medical resources; rationalize doctors’ working hours; and increase the number of oral healthcare professionals.

Periodontitis requires not only professional treatment and good patient adherence but also effective daily oral maintenance. In addition to tooth brushing, the use of interdental cleaning equipment at home can effectively prevent and control periodontal diseases [27]. In this study, 90% of patients self-reported brushing ≥ 2 times a day, 60% brushed for ≥ 3 min each time, and 66.4% used dental floss, oral irrigators or other cleaning devices, indicating that despite the low patient compliance, patients improved their awareness of oral hygiene after seeing a doctor, which is a good sign.

A family history of periodontitis is associated with SPT adherence, as also mentioned in this paper. However, there was no correlation observed between smoking and attitudes toward follow-up visits, which is inconsistent with the findings of previous studies [11]. This may be due to the large number of nonsmokers or former smokers in the study population. People who regularly used floss or oral irrigators had a more negative attitude toward follow-up visits, probably because they already had better oral hygiene habits and relatively less pronounced periodontal symptoms, that is, no obvious major complaints, which also echoes the points made above.

This study included advice taken from 108 participants, although not all of it was valid. Based on the results, a number of common suggestions were made: simplifying the visiting process and reducing the number of periodontal follow-up visits; increasing the weekend and nighttime availability of doctors in periodontal departments; ability to change online follow-up visits; having a person responsible for reminding patients of upcoming visits or using online tools such as mini-programs and public accounts to remind patients of appointment times; reducing treatment prices; increasing cooperation with online platforms to promote an understanding of periodontitis; and mobilizing social support from patients’ families. The above recommendations should be incorporated and referenced to improve compliance with future visits.

The current study has several limitations that need to be noted. First, the effectiveness of the telephone-based questionnaire was only 50.47%, with 481 patients refusing to answer the phone. One of the reasons may be the high level of distrust among Chinese people in revealing personal information over the phone; otherwise, a more representative set of findings could have been obtained. In addition, only one tertiary care hospital was investigated during this study, which covered most local periodontitis patients but could not span a wide geographical area and did not include private dental clinics or public general hospital dentistry departments. The level of self-efficacy for self-care may be a valid predictor of loss to follow-up [10], but the SESS questionnaire was not used in this survey, which would have made it more difficult given the length of the telephone survey. Therefore, larger and more diverse samples should be evaluated in future studies. This is an issue that the authors will address, as well as the possibility of considering combining prospective study surveys with telephone callbacks to minimize patient resistance regarding surveys.

## Conclusions

COVID-19 epidemic factors contributed to the loss to follow-up of periodontitis patients, but the personal factor of poor periodontal health awareness of the individual still remained the most important reason. Although the COVID-19 factor is no longer a threat at present, majority of patients have a negative attitude towards long-term SPT. Hence, patients who negatively toward compliance should be screened early, and targeted interventions need to be explored and implemented to reduce the loss to follow-up rate.

### Supplementary Information

**Additional file 1.**

**Additional file 2.**

## Data Availability

All data generated during this study are included in this published article’s Supplementary file 2.

## Abbreviations

SPT: Supportive periodontal therapy
AAP: American Academy of Periodontology
EFP: European Federation of Periodontology
PD: Pocket depth
CAL: Clinical attachment loss
BOP: Bleeding on probing
SESS: Self-efficacy scale for self-care
