# Supplementary material for: Impact of COVID-19 on patient follow-up during supportive periodontal therapy: a retrospective study based on phone call survey
Source: BMC Oral Health. 2023 Oct 28;23:811. doi: 10.1186/s12903-023-03539-1 (PMC10612285; doi:10.1186/s12903-023-03539-1)
Supplement: Supplementary file 1 — Additional file 1. [file 12903_2023_3539_MOESM1_ESM.docx]

Table 4 Explanation of each factor

| Variables | Specific content |
| --- | --- |
| Work factors | Too busy at work; Frequent business travel required |
| Family factors | Taking care of the family |
| Transportation or distance factors | Non-local patients; Poor access to public transportation to the hospital |
| Economic factors | Unable to afford periodontal treatment; Not covered by medical insurance |
| Personal factors | Self-perceived good periodontal condition; Forgot the follow-up time; Subjectively avoided treatment. |
| Physiological factors | Unable to tolerate the pain caused by the treatment; Pregnancy/lactation; Other medical conditions requiring treatment |
| Psychological factors | Dental Anxiety; Dental Fear |
| COVID-19 Epidemic factors | Fear of COVID-19 infection; local safety and epidemic prevention policies |
| Hospital factors | Large number of patients and long queues; Difficulty in making appointments; Changing to private dental clinics |
| Medical staff factors | No mention of follow-up made by medical staff; Not satisfied with the results of the doctor's treatment |
| Not stated / refused to answer | - |
